# Multidimensional Assessment of Eco-Innovation Implementation: Evidence from Spanish Agri-Food Sector

**DOI:** 10.3390/ijerph17041432

**Published:** 2020-02-23

**Authors:** Eva M. García-Granero, Laura Piedra-Muñoz, Emilio Galdeano-Gómez

**Affiliations:** Department of Economics and Business, University of Almería (Mediterranean Research Center on Economics and Sustainable Development, CIMEDES, Agrifood Campus of International Excellence, ceiA3), Ctra. Sacramento s/n, 04120 Almería, Spain; evaggranero@gmail.com (E.M.G.-G.); galdeano@ual.es (E.G.-G.)

**Keywords:** eco-innovation, implementation, multidimensional, agriculture, cluster analysis

## Abstract

Understanding eco-innovation is an essential endeavor to achieve global sustainable development. In this sense, further research on implementation is needed to expand knowledge beyond current boundaries. The aim of this paper is to contribute to this debate by conducting an original multidimensional analysis using Spanish agri-food sector data. The empirical methodology applies a combination of descriptive statistics, cluster analysis and the chi-squared test. Two groups of well-differentiated eco-innovative firms are identified, those with high and low eco-innovation implementation levels. Quality certifications, environmental consulting and cooperation with stakeholders are the variables that contribute most to distinguishing these two groups. The results also reveal that operating income volume, number of employees and commercialization volume are key factors to become more eco-innovative. In this sense, larger firms are found to have a higher level of eco-innovation implementation than small- and medium-sized enterprises. The main contributions of this work are fourfold. Firstly, it presents a comprehensive framework of eco-innovation implementation in its four dimensions (product, process, organizational and marketing). Secondly, it fills existing gaps in the literature by analyzing green organizational and marketing eco-practices. Thirdly, it expands the sectorial scope of eco-innovation research primarily focused on high-tech sectors. Finally, this study makes it possible to design certain policies for public and private decision makers.

## 1. Introduction

Eco-innovation (EI) is defined as the introduction of new products or significantly increasing a product/service’s value, improving processes, and creating organizational changes and new marketing solutions that can minimize the use of natural resources (including material, energy, water and soil), as well as reduce the release of dangerous substances throughout a product life cycle [1]. This concept plays a crucial role in the transition towards more sustainability development economies [2,3]. Furthermore, when dealing with damage caused to the environment, EI is especially important in contexts where it is necessary to introduce new, cleaner production techniques and provide more efficient products and changes in business models [4,5,6]. Therefore, identifying the main EI practices implemented by different sectors can help public and private decision makers to understand what instruments need to be developed for the purpose of promoting EI.

In recent years, a number of works on EI practices have been conducted. Although the Inter-American Development Bank recognizes that organizational and marketing EI practices are key points for developing more sustainable economies [7], most of the research conducted has focused only on product and process EI dimensions [8,9,10]. In these cases, the conclusions obtained do not accurately contemplate all eco-practices and can only provide a limited overview of the EI reality that exists within different sectors. Therefore, there are very few studies which provide a comprehensive framework for the analysis of EI in its main environmental dimensions (product, process, organizational and marketing), and they only contemplate a certain type of firm (e.g., small and medium size or multinationals) in non-European markets [11,12]. Furthermore, the vast majority of EI studies are focused on the industrial sector [13,14]. For this reason, it is necessary to expand the sectorial scope of this topic to develop more efficient green practices, regulations and policies. As Gente and Pattanaro [15] highlight, further research on EI implementation is needed to expand knowledge beyond current boundaries and achieve global sustainable development goals (SDGs). In this sense, despite the fact that they have received very limited attention, two sectors of great environmental importance are agriculture and exports [16,17,18]. In the case of the exports sector, exporting firms face a highly complex environment as they are more exposed to global competition [19,20]. Some researchers have highlighted that it is precisely for this reason that these companies are more likely to introduce EIs [21,22], especially those directly related to a sector with significant environmental impact such as agriculture. For this reason, among others, agricultural innovation is vitally important for the successful development of the food production sector as well as for preserving the environment [23,24].

This paper contributes to filling these gaps in the literature by developing a comprehensive framework for evaluating EI implementation multidimensionally. Therefore, it elaborates a frame of reference, which makes it possible to analyze the EI practices implemented in the Spanish wholesale sector of fresh fruits and vegetables and, in turn, identify the characteristics, variables and green dimensions that contribute to differentiate the most eco-innovative companies.

For this purpose, a combination of descriptive analysis, cluster analysis and the chi-squared test were utilized [25]. The statistical analysis reveals the existence of two groups of eco-innovative firms with distinct levels of EI. The differences between the two groups are highly dependent on operating income level, number of employees and volume of commercialization.

## 2. Theoretical Framework

Different EI frameworks have been suggested in the literature for analyzing the level of EI implementation. Kemp and Pearson [26] recommend the environmental technology, organizational, product/service and green systems dimensions of innovation. Carrillo-Hermosilla et al. [27] and Kiefer et al. [28] propose using the design, user, product-service, and governance dimensions of EIs. Moreover, Rodriguez-Rodriguez et al. [29] and Galdeano-Gómez et al. [30] point out the importance of EIs to achieve synergies between socio-economic and environmental dimensions in the agri-food sector. Furthermore, with the aim to standardize critical aspects of EI studies, the Eco-Innovation Observatory (EIO) [31] considers EI the “introduction of any new or significantly improved product, process, organizational change or marketing solution that reduces the use of natural resources and decreases the release of harmful substances across the whole life-cycle.” Following this guideline, some recent studies [11,12,32] propose four different main dimensions of EIs: product, process, organizational and marketing.

The present article builds upon the framework proposed by Marcon et al. [11] and García-Granero et al. [32] for analyzing EI implementation in an agri-food sector, because they provide a comprehensive overview of the main dimensions and subdimensions, accounting for the numerous individual characteristics of EI. In general, these four types of EI are complementary in many cases, so that the EI can be visualized with a holistic approach. Considering the close relationship with the environment that the agri-food activity has and the characteristics of companies (low-tech firms), the analysis of diverse dimensions can be important in order to offer a better view of EI implementation in this sector.

### 2.1. Eco-Innovation Dimensions

Product EI can be defined as the introduction of environmentally-friendly new products or significant improvements of product characteristics, such as advances in technical components and materials [33]. The theoretical framework on product EI is based on a vast line of research focused on the improvement of the type and quality of inputs used as well as on the sustainability of products with the aim of successfully complying with the current environmental regulations. Four main practices are contemplated in this approach. Some authors highlight the need to reduce the use of raw inputs in order to obtain less polluting products [14,34,35]. The use of cleaner materials or new inputs with lower environmental impact [7,8,9,10] is also proposed as a performance indicator. Marcon et al. [11] and Van Hemel and Cramer [36] analyze the use of recycled inputs. Besides, the product’s ability to be reused [9,10] is a practice examined to reduce the level of energy and materials consumed at the same time it decreases CO_2_ emissions and levels of waste [37].

According to Negny et al. [38], process EI modifies the organization’s operational processes and systems, decreases unit costs of production, produces new or significantly improved eco-products and reduces environmental impact. A wide range of EI literature investigates those practices in the process dimension that firms implement with the aim of reducing their negative environmental impact. Most of these investigations introduce water and energy consumption [8,39] as EI indicators. For example, Alkaya and Demirer [40] apply them in a review of the Turkish chemical industry, Catellacci and Lie [9] utilize them to analyze the manufacturing sector in Korea, and Rodríguez and Weingarten [10] in a study on the German industry sector. Other process EI indicators contemplated by researches, which ensure the efficient use of natural resources while optimizing the level of waste in the production and commercialization processes, are the reuse of components or materials [35] and their recycling [8,10,36]. Moreover, the eco-indicator number of patents [41,42,43,44] is introduced by some authors to measure EI. Although patents could be an output of company research efforts and investments, the latter are not always patented. For this reason, it is also necessary to include other indicators such as R&D expenditure [7]. Some authors emphasize the importance of analyzing the level of investment in R&D activities to gain a better understanding of EI [10,45,46]. The practical use of renewable energy and environmentally-friendly technologies is also a well-known eco-innovator and a large number of works address this topic. Frondel et al. [47] highlight the environmental benefit of introducing end-of-pipe technologies in the manufacturing process. Garrod and Chadwick [37] emphasize the importance of investing in clean technologies in a study on English companies. In the same vein, Guziana [48] defends the innovative proactivity of clean technology.

Organizational EI can be explained as new or significant improvements in routines, methods and actions that improve firms’ practices, relations and decisions with respect to the environment [11]. According to the findings of Chen [49], there are three types of green intellectual advantages, which encompass these essential corporate routines and practices [32]: human, structural and relational capital. Green human capital is attracting attention in the academic literature thanks to its impact on business decision-making. In this line, research such as Montalvo [50] and Chen and Chang [51] highlight the influence of green managerial characteristics in firm orientation towards an environmentally-friendly business model. Similarly, other authors uphold the role of senior staff in the green orientation business culture [52,53,54,55]. Furthermore, Banco Interamericano de Desarrollo (BID) [7] and Peng and Liu [56] underline the importance of introducing the analysis of a firm’s green human resources as an indicator which shows its innovative efforts. On the other hand, green structural capital includes organizational capabilities, organizational commitments, organizational culture and philosophies, patents, copyrights, etc. Environmentally-oriented culture is an eco-innovator that has been analyzed for more than two decades by an extensive body of research. According to Williams et al. [57], introducing environmental objectives into production plans and operations is a useful variable for analyzing EI level, while for authors such as Frosh and Gallopoulos [58] and Tibbs [59], the implementation of external environmental audits is a good indicator of a company’s intention of learning how to be more eco-innovative [60,61,62]. The hiring of environmental consulting services is another variable analyzed by the literature in this EI dimension [63,64,65]. In regard to green relational capital, the majority of the studies are focused on firm relationships with pressure groups [66,67,68] as a key factor to create new environmental improvement opportunities [49,69].

Marketing EI includes the implementation of new green marketing methods and refers to changes in product presentation, sales placement, communication, new methods of delivery, promotion or pricing strategies. Moreover, significant green changes in packaging are also considered important marketing EIs [11]. These innovation activities are relevant indicators for implementing and measuring EI as BID [7] emphasizes. However, marketing EI has received less attention than the other dimensions in environmental literature when analyzing the level of EI in a firm, sector or country [32]. The use of returnable packaging is the main practice studied by researchers [70,71,72], along with the use of recyclable packaging [36,73,74,75]. These green packaging design practices contribute towards reducing waste levels and the efficient use of resources [76,77]. What is more, biodegradable packaging is positioned as a key tool in several sectors to satisfy the environmental requirements of markets precisely because it is made of non-pollutant materials [78].

### 2.2. Eco-Innovation in the Agri-Food Sector

Innovation is positioned as a key factor in the discussion about the relation between agriculture and sustainability [79,80,81,82]. In fact, agricultural innovation is considered vital for the sustainability transition and achieving food security [83,84,85]. Thus, in recent years, some researches are focused on analyzing the EI phenom in this sector [16,86].

The increase in food crises, which place population health at risk, demands the implementation of new production practices that encourage the improvement of food safety levels. For this purpose, biological control and traceability implementation are two specific practices commonly carried out in the agri-food sector [87]. Barth et al. [86] point out the increment in product value that adds the traceability implementation. Galdeano-Gómez et al. [30] introduce the variable minimizing the use of fertilizers and phytosanitary product to measure the sustainability in the Spanish agricultural production. As a result, environmental sustainability is closely linked to biological control, as the latter is analogous to a high level of pest control [88].

Furthermore, the increase in population awareness about the environmental and health problems involved in the production and consumption of pollutant goods calls into question the need to use environmental certifications in order to achieve standards for safety and quality [86]. Certifications can be defined as a voluntary inspection process that audits and provides written assurance that a process, product or service meets a specific set of standards [89]. These standards prove the safety of the product customers consume [60,61,90]. In fact, there are several works that recommend the use of environmental certifications as an instrument for measuring EI [26,91,92,93]. Thus, private standards certifications, such as GLOBALG.A.P. (worldwide standard for Good Agricultural Practices) or GRASP (GLOBALG.A.P. Risk Assessment on Social Practices), are utilized in the European food sector as marketing tools to maintain consumer trust regarding the high quality of products, as well as to make considerations for animal welfare and environmental protection [94,95]. Recently, some studies introduced quality certifications in the EI analysis [93].

Other investigations highlight the importance of developing cooperation with stakeholders in the EI process [96,97]. Meanwhile, studies such us Drejeris and Miceikienè [98], Ulvenbland et al. [99] and Barth et al. [86] enhance the important value that a green organizational business model has in the transition towards sustainability. In this context, environmental attitudes, perceptions and intentions are included in the analysis of EI, and the staff environmental orientation is also a point of interest of investigations [86,98].

## 3. Materials and Methods

The research methodology was composed of the following main phases: a literature review, a survey questionnaire as a data collection tool, and, finally, a statistical data analysis including descriptive analysis, cluster analysis, and the chi-squared test. The three phases are detailed in the following section.

### 3.1. Definition of the Variables

A literature review based on Scopus and Web of Science (WoS) databases was conducted in order to identify contributions in the context of EI, not only to determine the variables, indicators and practices implemented, but also to identify what methodologies are applied to analyze EI.

García-Granero et al. [32] summarize the state of this field of research and highlight the main practices that have been taken into consideration by the literature to investigate how different sectors implement EI. This review determined which practices have a significant effect on the agri-food sector. Thus, the most relevant indicators and variables that should be measured were selected in order to analyze EI implementation in this sector (Table 1).

There is a common criterion throughout the EI literature for evaluating the level of product and process EI, regardless of the sector under analysis [8,9,10]. In the case of product EI, a great deal of the literature introduces variables that consider the improvement of the environmental characteristics of a product, either through the use of less polluting or reusable inputs [14,34,36]. As for process EI, most works analyze those variables related to the reuse, recycling or introduction of techniques that support the improvement of product quality [10,39]. However, with regard to organizational EI, while the vast majority of the EI studies in the last 20 years have focused on staff environmental culture and cooperation with stakeholders [52,58,100], other more recent studies have introduced practices such as environmental audits, environmental consulting or the implementation of environmental plans in daily business activity [7,26,62]. Similarly, while 20 years ago the EI literature did not contemplate marketing EI activities, subsequent studies highlight the introduction of green packaging and quality certifications as variables for measuring EI [39,60,61].

### 3.2. Sample and Data Gathering

**Figure 1:**
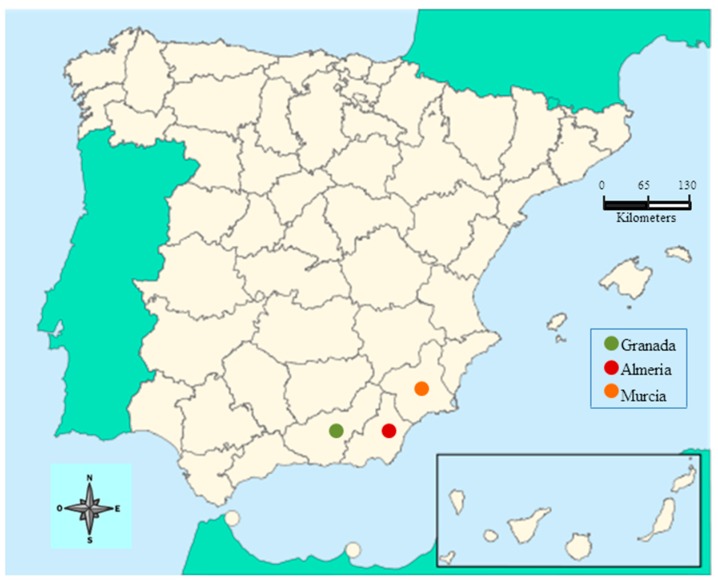
Location map of the region of Almeria, Granada and Murcia in Spain.

This study is focused on the Spanish agri-food sector, specifically in the southeast region (provinces of Almeria, Granada and Murcia), due to the increase of production in this area and the adaptation process of ecological practices required in consumer markets in recent decades [30]. In this case, agricultural activity has a strong impact on the environment because it involves intensive use of resources, requires intensive transport and generates a considerable amount of waste [101]. These negative externalities have implied a constant adoption of innovations and eco-efficiency methods of production and commercialization in the sector’s firms [93]. Moreover, Spain is the first exporter of fresh fruits and vegetables in the European Union and one of the three largest world exporters, together with the U.S. and China [102]. In terms of figures, Spanish exportation of these products reached 13.8 million tons in 2017, earning nearly 15 billion EUR [103]. In this context, the Spanish provinces of Almeria, Granada and Murcia contribute to these figures by more than 50 percent [95,104]. Figure 1 shows the location of this Spanish region.

To achieve the objectives proposed in this research, the data were obtained through surveys designed for this purpose. Questionnaires were aimed at staff who were closely involved in the EI aspects of the firms. All farming–marketing firms were contacted by telephone and all the individuals identified were then invited to participate in the survey via telephone or email. The survey was carried out in January and February 2019, coinciding with the production and commercialization season 2017–2018 (from September to July).

According to the Iberian Balance Sheet Analysis System (*Sistema de Análisis de Balances Ibéricos* in Spanish, SABI), 302 firms commercialized fruit and vegetables in the provinces of Almería, Murcia and Granada in 2017. The sample was randomly selected without replacement. The final number of valid surveys was 79. This represents a response rate of 22.32%, which is highly satisfactory. According to Menon et al. [105], the average top management survey response rate is in the range of 15–20 percent.

The descriptive analysis of the questionnaire responses from the sample of fresh fruit and vegetable commercialization companies is shown in Table 2.

This table indicates that limited liability companies dominate the sector (63.29%), followed by agrarian transformation companies (15.19%) and cooperatives (13.92%). The majority of the firms are between 15 and 30 years of age (43.04%). The figures also reveal that 39.24% of the firms have between 50 and 250 employees and 40.51% have operating incomes between 10 and 43 million EUR. Thus, these characteristics indicate that the sector is mainly represented by medium-sized companies. Furthermore, the survey also shows that 45.57% have a commercialization volume between 10 and 50 million kilos and 82.28% commercialize more than half of this volume in vegetables, meaning the sector is dominated by the fresh vegetable commercialization firms. In addition, 92.4% of the companies are European market oriented, as more than the half of their commercialized volume is allocated to this market.

### 3.3. Estimation Methods

Three statistical techniques were used: descriptive analysis, cluster analysis, and the chi-squared test [25,106,107]. Descriptive analysis provided a better understanding of the profile of companies in the sector. Cluster analysis is a multivariate statistical technique that is able to separate the sample into groups, achieving maximum homogeneity in each group and clearly differentiating between the groups. There are two main types of cluster analysis: non-hierarchical cluster and k-means cluster [108].

**Figure A1:**
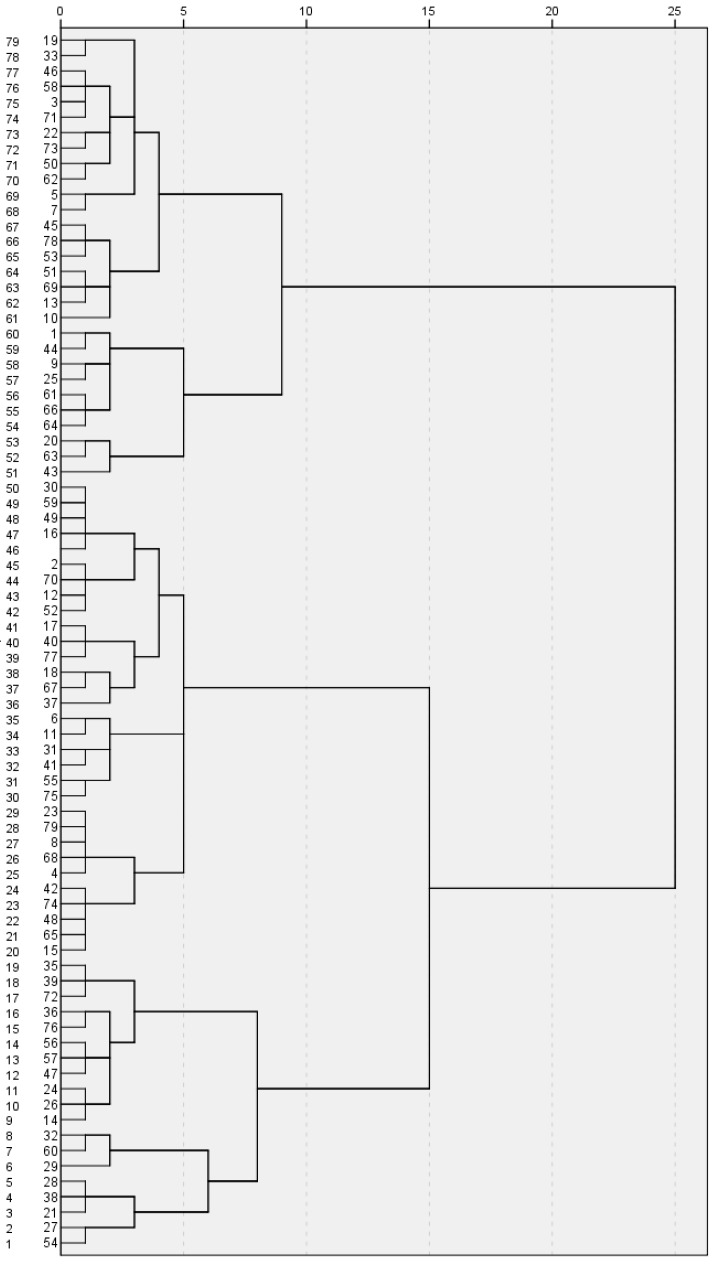
Cluster dendrogram.

Firstly, the non-hierarchical cluster analysis (Ward’s method) was used in this investigation to identify the number of groups that maximizes heterogeneity among them [109]. The results, presented in a dendrogram (see Appendix A, Figure A1), indicate that two is the optimal number of clusters in the sample: Group 1 (the lowest eco-innovator firms) and Group 2 (the highest eco-innovator firms).

Once the optimal number of groups was obtained, k-means cluster was applied, choosing the Euclidean distance as the distance measurement [110]. K-means cluster allocates every data point to the nearest cluster while keeping the centroids, previously calculated for each group, as small as possible. Next, a one-way ANOVA was carried out with the aim of testing the statistical differences between the clusters [25,111].

Finally, the chi-squared tests checked the relationship between the compositions of Groups 1 and 2 and the following profile variables: age of the company, operating income, number of employees, commercialization volume and percentage of commercialization volume in vegetables. The choice to use the chi-squared test was based on the relevance of knowing the main socio-economic factors that can affect firms’ decisions to implement EIs [25,112].

## 4. Results

The main results of applying descriptive statistics, cluster analysis and the chi-squared test are presented below.

### 4.1. Descriptive Statistics

Table 3 presents a brief description of the EI variables measured in the study in order to provide a profile of the firm’s eco-innovative level. Additionally, the correlation coefficients of variables are detailed in Appendix A (Table A1).

The data show that the average percentage of employees in charge of controlling and managing the quality of the products as well as the production processes is below 5.5%. This figure is rather small in relation to the maximum percentage of staff in these areas, which reaches 33% in some firms. Nevertheless, the mean level of green organizational culture displays a high value (3.73), which is reflected in the high implementation of certain eco-innovative practices, such as biological control, environmental consulting or production certified with GLOBALG.A.P.

These preliminary data also reveal the sector’s weakness in the implementation of some green practices, for example, recycling, the use of biodegradable packaging or the implementation of internal biological control laboratories. These practices display an average value below 0.5, which means a great deal must still be done to achieve greater environmental efficiency.

### 4.2. Cluster Analysis. Typology of Firms With Regards to Eco-Innovation Implementation

An exploratory factor analysis was conducted because the data collected were reported using a single informant from each company and from the same questionnaire in the same period [113]. Previously, variables were normalized with the aim of comparing different measuring instruments. The results reveal that the first factor captures only 28% of the variance, which demonstrates a low threat of common method bias. Next, a non-hierarchical cluster analysis (Ward’s method) was applied, prior to the k-means cluster analysis, in order to find the number of groups that maximize the differences between them, as mentioned in Section 3. The results obtained in the dendrogram (Appendix A, Figure A1) were analyzed and two clusters appeared as the best solution. In order to confirm the number of clusters selected, the Calinski test was performed. The two-group solution with a Calinski–Harabasz pseudo-*F* value of 87.44 was largest, indicating that the two-group solution was the most distinct compared with the three-group (72.85), four-group (55.60) and five-group (48.03) solutions. Thus, two different groups were identified: Group 1, consisting of firms with a lower level of EI implementation; and Group 2, made up of firms with a higher level of EI implementation. The results are shown in Table 4, which displays the values of the main variables.

Table 4 also shows the analysis of the variance of the cluster analysis (one-way ANOVA analysis). All the variables, except “the use of biodegradable packaging,” differ statistically between groups with a level of likelihood of 5% (*p*-value < 0.05). The results also reveal that the variables “number of quality certifications,” “percentage of GLOBALG.A.P. certified hectares” and “percentage of GRASP certified hectares” are those that contribute most to the differentiation between groups; followed by the variables “biological control,” “environmental consulting” and “cooperation with stakeholders.”

**Figure 2:**
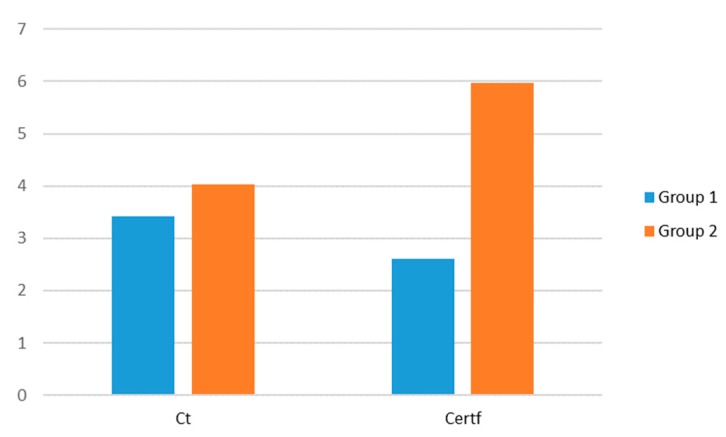
Average scores for Groups 1 and 2 in eco-innovations quantitative variables measured in number scale. Notes: Ct = Environmentally-oriented culture; Certf = Quality certifications.

**Figure 3:**
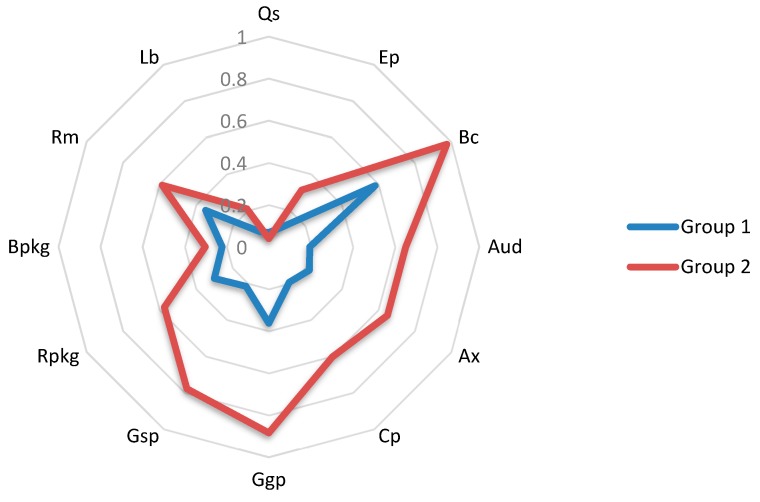
Average scores for Groups 1 and 2 in eco-innovations quantitative variables measured in percentage scale and qualitative variables. Note: Ep = Ecological/integrated production; Bc = Biological control; Rm = Recycled/reused materials; Ct = Environmentally-oriented culture; Qs = Quality staff; Lb = Analysis laboratory; Aud = Environmental audit; Ax = Environmental consulting; Cp = Stakeholders cooperation; Certf = Quality certifications; Ggp = GLOBALG.A.P. certification; Gsp = GRASP certification; Rpkg = Recycled packaging; Bpkg = Biodegradable packaging.

Figure 2 presents the mean values of Groups 1 and 2 for the different quantitative variables measured on a numerical scale shown previously in Table 1. In contrast, Figure 3 displays the mean values of Groups 1 and 2 for the different qualitative variables and those quantitative variables measured on a percentage scale.

Group 2 is comprised of firms with higher environmental cultures. This orientation leads them to introduce environmental plans and aims into their daily activities. Also, the senior staff place the utmost importance on all company operations being environmentally respectful and fulfilling the environmental goals established. Thus, these firms conduct environmental audits and cooperate with environmental experts, universities and R&D groups in order to discover new ways to reduce their negative environmental impact. Moreover, these firms comply with a large number of certifications in order to meet the quality standards requested by consumers and markets. Furthermore, some firms in Group 2 have introduced internal laboratories with the aim of conducting random pesticide and insecticide controls to ensure levels of these inputs are kept to the minimum. Finally, their use of recyclable and biodegradable packaging is higher.

As for Group 1 companies, in the survey they also responded as having a high level of environmentally-oriented culture and display a slightly higher percentage of employees in the quality department than Group 2. Nevertheless, the results reveal low values of EI implementation in practices such as the number of quality certifications and the volume of ecological production commercialized. In addition, their cooperation with stakeholders as well as their use of recyclable packaging and their recycled material volume is still far from Group 2 implementation levels.

### 4.3. Chi-Squared Tests

In order to understand how and why the two groups are different, a chi-squared analysis was used to determine which characteristics in the two clusters differ [112]. The chi-squared test examines the relationship between the composition of groups and the following profile variables: age of the company, percentage of the commercialization volume in vegetables, operating incomes, number of employees and commercialization volume. With an error of less than 5%, the analysis revealed that the age of the company and the percentage of the commercialization volume in vegetables are not factors that contribute to differentiating the level of EI between groups, as shown in Table 5 and Table 6.

Table 7 presents the observed and expected frequencies for the operating income in Groups 1 and 2. The observed number of firms in Group 1 with operating incomes under 43 million EUR is higher than the expected frequency, while the observed number of firms in Group 2 with operating incomes above 43 million EUR is higher than the expected number. Thus, those firms whose operating incomes are above 43 million EUR are influenced by factors that drive them to be more eco-innovative.

Table 8 and Table 9 present the observed and expected frequencies for the number of employees and the millions of kilos commercialized in Groups 1 and 2, respectively. The observed number of firms in Group 2 with more than 250 employees and a volume of commercialization over 50 million kilos is higher than the expected number. Therefore, firms with more than 250 employees and a volume of commercialization over 50 million kilos are influenced by factors that drive them to be more eco-innovative.

## 5. Discussion

The statistical results highlight some weaknesses in EI implementation in the Spanish agri-food sector. On one hand, the sector does not place enough importance on the implementation of certain eco-innovative practices (e.g., waste level, water/energy consumption or R&D investments). Consequently, it also ignores other EI practices that are very important to achieve cleaner production and environmental sustainability in the sector.

On the other hand, regarding product EI, despite the fact that ecological and integrated production has increased in recent years, it continues to be lower than that of traditional production. As the cluster analysis demonstrates, the level of ecological or integrated production does not reach 50% of the total production. Regarding process EI, although all the sector companies implement traceability control due to its being legally required, biological control is not implemented by the whole sector, despite being a key factor in quality control of goods and ecosystems. These results demonstrate the need to implement eco-support policies along with more mandatory environmental policies, with the aim of urging companies to introduce eco-innovative practices in their daily activities. Environmental regulations are positioned as key drivers of EI initiatives [36] and have special influence on Spanish firms [114].

Concerning organizational EI, Group 2, which is comprised of the most eco-innovative firms, has a greater propensity to establish relationships with environmental experts and stakeholders in order to improve its environmental impact. As the descriptive analysis highlights, this group of companies not only regularly performs environmental audits and requests environmental consulting, but it also has a higher number of staff allocated to control the quality of goods and the production process. This description confirms the conclusion reached by González-Moreno et al. [115] regarding the need to create intense relationships with stakeholders in order to develop a fluent EI process in the food sector. Also, these findings are in line with other works that underscore the importance of relationships with pressure groups in the development of EI [28,65,116,117].

Regarding marketing EI, green packaging design is another point to address. The use of recycled or biodegradable materials is positioned as an environmental solution, but despite increased usage in recent years, its implementation is still low [78]. As the cluster analysis reveals, the use of recycling packaging is located far below 40% in Group 1 and the use of biodegradable packaging in both groups barely reaches 30%. Thus, in accordance with the recommendation of Ahmed and Alam [118], promoting the use of green packaging is an important environmental and marketing tool. In environmental terms, it contributes significantly to reducing waste levels and CO2 emissions; while in terms of marketing, it contributes to market growth. Futhermore, as Verghese and Lewis [119] defend, cooperation in packaging systems ensures reductions in costs and increases in environmental efficiency. With regard to environmental certifications, they are a tool that is increasingly implemented by the sector and the indicators related to them contribute most to differentiating the EI level between groups, as the ANOVA analysis reveals. According to the findings of Segarra-Oña et al. [89], these certifications are indicative of incremental innovations.

The results also reveal that most of the sector companies are small- and medium-sized companies (75.9%); however, those companies with an income volume above 43 million EUR are more likely to implement eco-innovative practices. This is in line with Becheikh et al. [11], who point out that the innovation activity is more probable in large-sized firms. As Arranz et al. [120] states, the lack of EI development in firms can be caused by the perception of high costs, the need for financing and the lack of environmental knowledge. In this line, implementing policies that promote financial incentives as well as non-financial, such as seeking environmental partners, is a key factor to achieve cleaner production [115,120,121,122]. In accordance with the findings of Ghisetti and Pontoni [123], regulatory stringency has positive, significant effects on EI, and policy-makers need to introduce regulatory-standards in order to further promote sustainable transition. This is of great interest especially in the agri-food sector, highly linked to the use of natural resources and the food value chain. According to the SDGs, promoting EI the in agri-food sector contributes to encouraging companies to implement greener production methods with less amount of waste, use natural resources in an efficient way and obtain products more respectful to the public health, in accordance with the quality requirements [11,29,124].

## 6. Conclusions

This study conducts a multidimensional analysis of EI implementation. For that purpose, the study complies sets of variables for the four main dimensions of EI (product, process, organizational and marketing), utilizing data from a survey carried out ad hoc on the agricultural sector in the southeast of Spain. Thus, seeking to undertake much more than a mere conventional analysis of EI implementation and to expand the sectorial focus of study, the empirical analysis examined several types of EI practices implemented in an agri-food sector: Spanish wholesalers of fruits and vegetables.

The statistical analysis reveals that, despite having a group of more eco-innovative companies, the efforts made to reduce negative environmental externalities are mostly limited to large companies as they have more economic resources. In fact, the vast majority of the sector is composed of small-and medium-sized companies, which show less propensity to eco-innovate, especially in those green practices with higher costs of implementation, such as the use of recyclable and biodegradable packaging or the implementation of internal analysis laboratories for better control of pollutant input usage in the production of the goods. Moreover, although most of the companies are certified with quality certifications, not all of their production comes from farmers that are standard certified. In addition, regarding the group of less eco-innovative firms, the results highlight the need for an increase in their environmental awareness. For instance, although they responded in the survey as introducing environmental plans and aims in their daily activity, the insufficient degree of cooperation with environmental experts reveals that a great deal of work still remains to be done in order to achieve a sustainable production process. These results demonstrate the need to develop new financial and non-financial regulations that support innovation practices in the sector, especially for small- and medium-sized companies, while also taking into consideration the importance of organizational and marketing eco-dimensions.

### 6.1. Implications for Theory and Practice

Overall, this investigation develops a comprehensive framework for a multidimensional analysis of EI implementation in its four dimensions, filling the gap in the literature, which has focused mainly on analyzing product and process, and only includes organizational and marketing EI to some extent. Also, as most of the analyses of this issue are focused on the industrial sector, this research offers a new framework on the state of EI implementation in a high impact environmental sector: the agri-food sector. Thus, this study makes it possible to broaden the focus of analysis and develops a method of EI analysis that more closely resembles reality.

In addition, the findings of this research infer some policy implications for both public and private decision makers, contributing to the transition towards sustainable development. On one hand, it allows governments to know in which directions regulatory efforts should be focused. For example, they should promote more fiscal benefits and economic aid to encourage small- and medium-sized companies to implement greener practices, especially related to organizational and marketing dimensions. Small- and medium-sized companies have to make more efforts to bear the high costs of implementing eco-practices, so facilitating R&D cooperation with universities and research centers would support the assumption of these costs. In addition, decision makers should encourage the access of these types of companies to public funds specially destined for the development of ecological practices. On the other hand, it provides companies with knowledge on green practices that can be implemented to become more environmentally efficient, and also helps them to understand the importance of implementing EI practices in all dimensions in order to achieve cleaner production and develop sustainable production processes.

### 6.2. Limitations and Future Research

Like all empirical research, this study features some limitations, which could serve as reference for future works. Firstly, some relevant EI variables could not be measured (e.g., level of waste or recycling of materials) because the firms simply do not keep logs on certain data. Therefore, firms should be encouraged to register this important information, which would allow future works to focus on expanding the variables that have an influence on EI implementation. Secondly, a posterior EI analysis could be conducted to compare results with those initially obtained to determine their evolution over time. Thirdly, the study focuses on the Spanish agri-food sector, so it would be particularly interesting if future research conducted a similar analysis of other national and international agri-food sectors in order to make comparisons. Finally, the multidimensional assessment framework of EI implementation proposed by this paper could be applied to other sectors.

## Figures and Tables

**Table 1:** Indicators and variables of eco-innovations included in the analysis.

Eco-Innovation Dimension	Eco-innovation Indicator	Description of the Variable		
		Name	Survey Question	Measurement Scale
Product EI	Ecological/integrated production	Ep	What percentage of the total production is dedicated to ecological/integrated production?	Percentage
	Biological control	Bc	Has your firm implemented biological control?	Dichotomous scale

Process EI	Recycled/reused materials	Rm	What percentage of the total use of plastics, pallets and packaging is recycled or reused?	Percentage
			What is the importance of your company’s environmental impact?	
			What is the importance of adopting environmental plans and objectives in the company?	
			What is the importance of achieving the environmental plans and objectives adopted?	
	Environmentally-oriented culture	Ct *	What is the importance of staff working with respect for the environment?	Likert scale (1–5)
			What is the importance of investing in environmental initiatives?	
Organizational EI			What is the importance of implementing EIs?	
	Quality staff	Qs	Percentage of employees working in the quality department?	Percentage
	Analysis laboratory	Lb	Does your firm have an internal analysis laboratory?	Dichotomous scale
	Environmental audit	Aud	Does your firm perform environmental audits?	Dichotomous scale
	Environmental consulting	Ax	Does your firm request environmental consulting from any expert?	Dichotomous scale
	Stakeholder cooperation	Cp	Does your firm cooperate with universities or R&D centers?	Dichotomous scale
		Certf	Number of quality certifications?	Natural numbers
	Quality certification	Ggp	Percentage of hectares certified with GLOBALG.A.P. certification?	Percentage
Marketing EI		Gsp	Percentage of hectares certified with GRASP certification?	Percentage
	Green design packaging	Rpkg	Use of recycled packaging?	Percentage
		Bpkg	Use of biodegradable packaging?	Dichotomous scale

* This variable is given by the average from the six survey questions. Source: own elaboration.

**Table 2:** Profile of the final sample (frequencies for descriptive variables).

Variable	Description	Frequency (*N* = 79)
Age (years)	<15 15–30 31–45 >45	31 34 9 5
Number of employees	<50 50–250 >250	25 31 23
Legal form	Limited liability companies (SL in Spanish)	50
	Anonymous society (SA in Spanish)	6
	Agrarian transformation company (SAT in Spanish)	12
	Cooperatives	11
Operating income (million EUR)	<10 10–43 >43	28 32 19
Commercialization volume (million kg)	<10 10–50 51–100 >100	27 36 8 8
Percentage of commercialization volume in vegetables (%)	<50 ≥50	14 65
Percentage of commercialization volume allocated to European market (%)	<50 ≥50	6 73

**Table 3:** Summary statistics for the EI variables in the sample.

Variable	Variable Name	Mean	Std. Dev.	Min.	Max.
Product EI					
Ep	Ecological/integrated production	0.21	0.33	0	1
Process EI					
Bc	Biological control	0.80	0.40	0	1
Rm	Recycled/reused materials	0.47	0.37	0	1
Organizational EI					
Ct	Environmentally-oriented culture	3.73	0.86	0	5
Qs	Quality staff	0.053	0.37	0	0.33
Lb	Analysis laboratory	0.15	0.36	0	1
Aud	Environmental audit	0.44	0.50	0	1
Ax	Environmental consulting	0.46	0.50	0	1
Cp	Stakeholder cooperation	0.42	0.49	0	1
Marketing EI					
Certf	Quality certifications	4.44	2.57	0	11
Ggp	GLOBALG.A.P. certification	0.64	0.36	0	1
Gsp	GRASP certification	0.52	0.41	0	1
Rpkg	Recycled packaging	0.44	0.38	0	1
Bpkg	Biodegradable packaging	0.27	0.44	0	1

**Table 4:** Characteristics of identified clusters and test statistics of one-way ANOVA.

		Eco-Innovative Firm Clusters					
		Group 1 *N* = 37		Group 2 *N* = 42			
		Low		High			
Variable	Variable Name	Mean	Std. Dev.	Mean	Std. Dev.	*F*	*p*-Value
Product EI							
Ep	Ecological/integrated production	0.10	0.24	0.31	0.37	9.57	.003
Process EI							
Bc	Biological control	0.58	0.50	0.98	0.15	22.29	0.000
Rm	Recycled/reused materials	0.35	0.33	0.59	0.37	10.80	0.002
Organizational EI							
Ct	Environmentally-oriented culture	3.42	0.87	4.03	0.65	13.65	0.000
Qs	Quality staff	0.07	0.08	0.04	0.05	3.46	0.067
Lb	Analysis laboratory	0.08	0.27	0.30	0.41	2.74	0.002
Aud	Environmental audit	0.19	0.42	0.65	0.49	17.33	0.000
Ax	Environmental consulting	0.22	0.44	0.65	0.49	14.70	0.000
Cp	Stakeholders cooperation	0.19	0.40	0.60	0.50	17.96	0.000
Marketing EI							
Certf	Quality certifications	2.61	1.91	5.98	1.85	69.63	0.000
Ggp	GLOBALG.A.P. certification	0.36	0.35	0.88	0.16	71.56	0.000
Gsp	GRASP certification	0.22	0.30	0.78	0.28	82.28	0.000
Rpkg	Recycled packaging	0.30	0.37	0.57	0.36	10.79	0.002
Bpkg	Biodegradable packaging	0.22	0.41	0.30	0.47	0.87	0.355

**Table 5:** Observed and expected frequencies for age of the company in Groups 1 and 2.

Age of the Company (Years)			Less than 15	Between 15–30	Between 30–45	More than 45	Total
Group	1	Observed	17	17	2	1	37
		Expected	14.5	15.9	4.2	2.3	37
	2	Observed	14	17	7	4	42
		Expected	16.5	18.1	4.8	2.7	42

Pearson chi-squared: 4.570; *df* = 3; *p* = 0.206.

**Table 6:** Observed and expected frequencies for percentage of the commercialization volume in vegetables in Groups 1 and 2.

Percentage of the Commercialization Volume in Vegetables (%)			Less than 50	More than 50	Total
Group	1	Observed	8	29	37
		Expected	6.6	30.4	37
	2	Observed	6	36	42
		Expected	7.4	34.6	42

Pearson chi-squared: 0.726; *df* = 1; *p* = 0.394.

**Table 7:** Observed and expected frequencies for operating income in Groups 1 and 2.

Operating Income (Thousands of Euros)			Less than 10,000	Between 10,000–43,000	More than 43,000	Total
Group	1	Observed	21	16	0	37
		Expected	13.1	15	8.9	37
	2	Observed	7	16	19	42
		Expected	14.9	17	10.1	42

Pearson chi-squared: 25.787; *df* = 2; *p* = 0.000.

**Table 8:** Observed and expected frequencies for number of company employees in Groups 1 and 2.

Employees (number)			Fewer than 50	Between 50-250	More than 250	Total
Group	1	Observed	18	19	0	37
		Expected	11.7	14.5	10.8	37
	2	Observed	7	12	23	42
		Expected	13.3	16.5	12.2	42

Pearson chi-squared: 29.221; *df* = 2; *p* = 0.000.

**Table 9:** Observed and expected frequencies for commercialization volume in Groups 1 and 2.

Commercialization volume (millions of kilos)			Fewer than 10	Between 10–50	Between 50–100	More than 100	Total
Group	1	Observed	16	20	0	1	37
		Expected	12.2	16.9	3.7	4.2	37
	2	Observed	10	16	8	8	42
		Expected	13.8	19.1	4.3	4.8	42

Pearson chi-squared: 15.017; *df* = 3; *p* = 0.002.

## Appendix A

**Table A1:** Pairwise correlation coefficients of variables.

	Ct	Ep	Bc	Qs	Aud	Ax	Cp	Certf	Ggp	Gsp	Rpkg	Bpkg	Rm	Lb
**Ct**	1	0.149	0.084	0.068	0.012	0.034	−077	−243	−333	−255	−203	−052	−178	0.400
**Ep**	0.149	1	0.187	0.155	0.257	0.295	0.311	0.273	0.351	0.337	0.156	0.172	0.103	0.223
**Bc**	0.084	0.187	1	0.253	0.408	0.113	0.038	0.256	0.140	0.226	0.150	0.095	0.020	0.105
**Qs**	0.068	0.155	0.253	1	0.259	0.208	0.044	0.383	0.381	0.334	0.188	0.161	0.228	0.126
**Aud**	0.012	0.257	0.408	0.259	1	0.465	0.278	0.474	0.291	0.231	0.163	0.156	0.024	0.120
**Ax**	0.034	0.295	0.113	0.208	0.465	1	0.256	0.339	0.206	0.152	0.112	0.082	0.009	0.250
**Cp**	−077	0.311	0.038	0.044	0.278	0.256	1	0.436	0.271	0.275	0.107	0.013	0.147	0.357
**Certf**	−243	0.273	0.256	0.383	0.474	0.339	0.436	1	0.491	0.473	0.399	0.165	0.191	0.134
**Ggp**	−333	0.351	0.140	0.381	0.291	0.206	0.271	0.491	1	0.535	0.343	0.018	0.298	0.038
**Gsp**	−255	0.337	0.226	0.334	0.231	0.152	0.275	0.473	0.535	1	0.334	0.141	0.250	0.031
**Rpkg**	−203	0.156	0.150	0.188	0.163	0.112	0.107	0.399	0.343	0.334	1	0.178	0.408	−081
**Bpkg**	−052	0.172	0.095	0.161	0.156	0.082	0.013	0.165	0.018	0.141	0.178	1	0.175	−095
**Rm**	−178	0.103	0.020	0.228	0.024	0.009	0.147	0.191	0.298	0.250	0.408	0.175	1	−022
**Lb**	0.400	0.223	0.105	0.126	0.120	0.250	0.357	0.134	0.038	0.031	−081	−095	−022	1
